# Clinical study on the treatment of male infertility with Wuwei Fuzheng Yijing decoction based on microplastics: Study protocol for a randomized controlled trial

**DOI:** 10.1097/MD.0000000000031265

**Published:** 2022-10-14

**Authors:** Miaomiao Ma, Baojun Ju, Xiao Li, Junchao Yao, Luyu Li, Yongtao Zhang, Shuotong Tang, Chenming Zhang

**Affiliations:** a Henan University of Chinese Medicine, Zhengzhou, China; b The First Affiliated Hospital of Henan University of Chinese Medicine, Zhengzhou, China; c The Second Clinical Medical School, Henan University of Chinese Medicine, Zhengzhou, China.

**Keywords:** male infertility, microplastics, semen, traditional Chinese medicine

## Abstract

Environmental pollution and male infertility have become global public health problems. The presence of microplastics (MPs) has been detected in the human body, and it has also been demonstrated that MPs can cause damage to the reproductive system. Wuwei Fuzheng Yijing Decoction (WWFZYJ) is effective in treating male infertility. Therefore, we designed a clinical randomized controlled trial to observe the effect of WWFZYJ on the content of MPs and semen quality in male infertility patients, and to evaluate its security.

**Methods:**

In this randomized controlled study, 66 eligible patients were randomly assigned in a 1:1 ratio to a treatment group (WWFZYJ Decoction) and a control group (Coenzyme Q10 tablets combined with vitamin E soft capsules) for 8 weeks. The content of MPs in semen, sperm DNA Fragmentation Index (DFI), and semen analysis (including sperm density, sperm count, forward motile sperm, sperm motility, etc) will be used as primary indicators, and Traditional Chinese Medicine (TCM) syndrome scores will be used as secondary indicators. Vital signs (such as respiration, heart rate, body temperature, blood pressure, electrocardiogram, etc), blood routine, urine routine, stool routine, liver function, and renal function will be used as safety indicators. The primary and secondary indicators will be performed at 0th and 8th week, and the safety indicators will be performed at 0th, 4th, and 8th week.

**Discussion:**

This study will provide evidence for the efficacy and safety of WWFZYJ in treating male infertility and reducing the content of MPs in semen, and further explore the effects of MPs on male fertility.

## 1. Introduction

Fertility is an important factor affecting social development. In recent years, with the change of people’s lifestyle and the aggravation of environmental pollution, the number of infertility patients has continued to increase worldwide, and the problem of male infertility caused by environmental pollution has also been paid more and more attention. The causes of infertility include male infertility and female infertility, of which male factors account for 20% to 70%.^[1]^ The “Research Report on the Status of Infertility in China” pointed out that in the past 20 years, the infertility rate has increased from 3% to 12.5%, and the quality of male sperm is declining.^[2]^ Plastic products are widely used in public life, and up to 80% of plastic products are directly discharged into the environment without effective treatment. These plastics are difficult to degrade, and the degradation cycle can last for hundreds of years. The existing garbage disposal methods can only crack the plastics into countless microplastics (MPs) smaller than 5 mm. In addition, we are often exposed to MPs in our daily life, such as cosmetics, toothpaste, detergents, etc. MPs are a growing global problem due to their abundance in the near term and persistent toxicity to future generations.

MPs can enter the human body through the food chain, and the presence of MPs has been detected in the human lung,^[3]^ intestine,^[4]^ and placenta.^[5]^ Studies have found that MPs can cause reproductive system damage in experimental animals. Amereh et al^[6]^ found that PS nanoplastics with an average diameter of 38.92 nm were administered orally at doses of 1, 3, 6, and 10 mg/kg/day, respectively. After 5 weeks of rats, sperm DNA Fragmentation Index (DFI) can be increased to different degrees. Trifuoggi et al^[7]^ found that the sperm pronuclear damage caused by MPs to star sea urchins can be passed on to offspring. Recent studies have shown that the minimum human equivalent dose of MPs that can cause abnormal semen quality in men is 0.016 mg/kg/d.^[8]^

The efficacy of Wuwei Fuzheng Yijing (WWFZYJ) Decoction in the treatment of male infertility has been proved to be satisfactory through clinical trials.^[9]^ The influence of semen analysis-related parameters, to further explore the influence of MPs on the semen quality and reproductive function of male infertility patients, to find a new entry point for the treatment of male infertility, so as to further explore and provide new insights into male infertility caused by environmental pollution ideas.

## 2. Methods and analysis

### 2.1. Design

**Figure 1.**
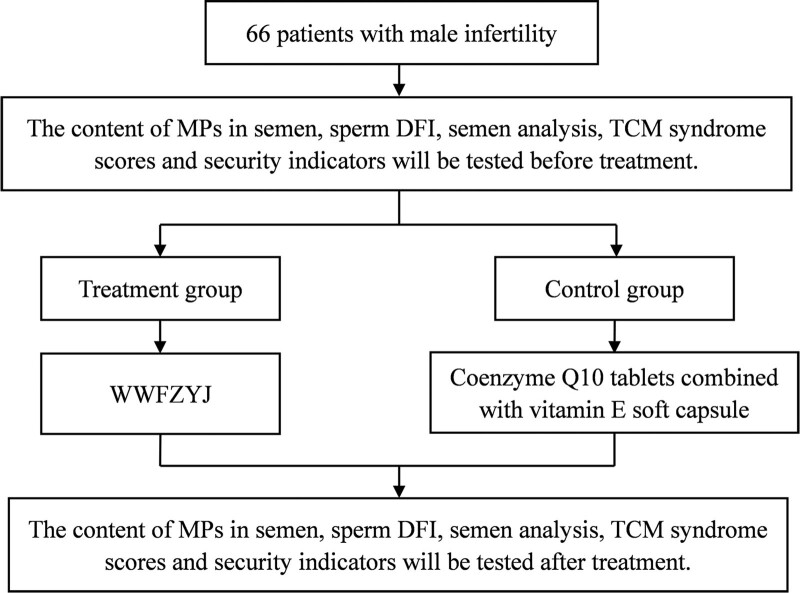
Research flow chart.

We are conducting an 8-week, single-center, rater-blinded, randomized controlled trial. The treatment group (WWFZYJ Decoction) and the control group (Coenzyme Q10 tablets combined with vitamin E soft capsules) were compared to observe the effect of WWFZYJ on the content of MPs and semen quality in male infertility patients, and to evaluate its safety. The trial will be carried out in the Andrology Department of the First Affiliated Hospital of Henan University of Chinese Medicine. All participants were required to provide written informed consent prior to entry into the trial. The research flow chart is shown in Figure 1.

### 2.2. Ethic approval

This protocol was carried out in accordance with the principles of the Declaration of Helsinki,^[10]^ and it has been approved by the Ethics Review Committee of the First Affiliated Hospital of Henan University of Chinese Medicine (approval number: 2022HL-333-02) and the whole process was followed up. We registered the study in Chinese Clinical Trials Registry (registration number ChiCTR2200063340).

### 2.3. Participants

After obtaining informed consent, this study will include 66 male infertility patients. We will make promotional posters in the andrology clinic area or release information through social media of hospital to recruit participants, and participants who meet the research criteria will be invited to the Andrology Department of the First Affiliated Hospital of Henan University of Chinese Medicine for the research. Eligible participants will be randomized 1:1 to treatment and control groups. The treatment will last 8 weeks.

#### 2.3.1. Diagnostic criteria.

The diagnostic criteria of male infertility will be formulated with the relevant diagnostic criteria of the “Expert Consensus on Male Reproductive Genetics Examination” compiled by the Andrology Branch of the Chinese Medical Association. Specifically: Couples of childbearing age, with normal sexual life and no contraceptive measures, the woman’s reproductive capacity is normal, and the woman cannot conceive naturally within 1 year due to the man’s factors, which is called male infertility; perm DFI ≥ 30% means poor sperm DNA integrity.

#### 2.3.2. Inclusion criteria.

The inclusion criteria are as follows: Meet the above diagnostic criteria; Male between the ages of 25 to 50; Abstinence time is 2 to 7 days; All patients had not taken any infertility medication within 1 month prior to treatment; Willing to participate in this research and sign the informed consent.

#### 2.3.3. Exclusion criteria.

The exclusion criteria are as follows: Abnormal semen due to chromosomal factors; Azoospermia; Not abstinence within the specified time; Abnormal semen parameters caused by reproductive tract infection or genitourinary system and other related diseases, such as spermatic vein Varicose; Those who have taken drugs that hinder sperm production and sperm motility within 3 months, or those who have received radiotherapy and chemotherapy, have serious primary diseases, and patients with mental illness; Those who have a history of allergies to therapeutic drugs; Based on research From the perspective of the subject, the subject may have other conditions that affect the clinical trial.

### 2.4. Sample size

Sample size power calculation was performed using PASS 15.0 software, with *α* = 0.05 (2-tailed test). The sample size of the experimental group is N_1_, and the sample size of the control group is N_2_. It is assumed that N_1 _= N_2_ = 30. Referring to the current statistics on the clinical efficacy of traditional Chinese medicine (TCM) in the treatment of male infertility, it is proposed that the effective rate of the treatment group is *μ*_1_ = 80%, and that of the control group is 80%. The efficiency rate is *μ*_2_ = 50%, and the standard deviation *σ* is 1 to 10. The power calculation results showed that under the condition that *σ* is 1 to 10, 100% power = 1.00000 can be achieved, rejecting the null hypothesis that the mean difference between the 2 groups are equal. The sample size included in this study met the criteria for follow-up studies. Considering the dropout of cases and assuming a loss-to-follow-up rate of 10%, the final sample size is 66 cases, with 33 people in the treatment group and 33 in the control group.

### 2.5. Randomization and blinding

This study will use random assignment concealment. Participants who provided informed consent and met the inclusion criteria were randomly assigned in a 1:1 ratio to treatment and control groups. Randomization will be based on random numbers generated by the computer. Independent researchers will prepare the distribution in an opaque envelope containing the distribution sequence number and are responsible for concealing the distribution sequence. Due to the specificity of the intervention, double-blinding was impossible. Outcome assessors, data curators will be unaware of treatment assignments.

## 3. Interventions

### 3.1. Basic interventions

The 2 groups were given basic interventions, including instructing patients to quit smoking and alcohol during taking the medicine, prohibiting sauna and basin (pool) bathing, avoiding exposure to high temperature, radiation pollution, chemical poison pollution and other environments as much as possible, regular work and rest during treatment, and relaxation.

### 3.2. Drug intervention

#### 3.2.1. Treatment group.

This group of patients will take WWFZYJ granules orally. WWFZYJ consists of: astragalus 30g, wolfberry 15g, achyranthes 15g, schisandra 15g, and plantain 12g. The granules were uniformly purchased from the Chinese Pharmacy of the First Affiliated Hospital of Henan University of Chinese Medicine and produced by Sichuan Xinlu Pharmaceutical Technology Development Co., Ltd. (Sichuan, China). 1 bag each time, 1 time in the morning and 1 time in the evening, dissolved in boiled water and taken orally, the course of treatment is 8 weeks.

#### 3.2.2. Control group.

This group of patients will take vitamin E soft capsules combined with coenzyme Q10 tablets. Vitamin E soft capsules were provided by Zhejiang Pharmaceutical Co., Ltd., approved by Chinese medicine H20003539, 1 capsule each time, 3 times a day, orally; Coenzyme Q10 tablets were provided by Eisai (China) Pharmaceutical Co., Ltd., approved by Chinese medicine H10930021, each 1 tablet, 3 times a day, orally. The course of treatment is 8 weeks.

### 3.3. Combined treatment regulations

If the subjects meet the inclusion criteria, and the patients with other diseases need to continue to use the drug in the clinical trial, or if they really need to add other drugs or treatment methods due to the needs of the disease treatment, the drugs used should be recorded in the case report form (CRF) in detail. name (or treatment method), dosage, frequency of administration, time of administration, etc. The drugs and treatment methods that must be taken in combination with other diseases must be recorded in detail in the combined medication table. If disease progresses during the study, participants can withdraw from the study and use other treatments. The patient will be asked to complete relevant examinations and assessments as much as possible, and the case will be considered an excluded case.

## 4. Outcome measures

### 4.1. Primary outcome

MPs content in semen, sperm DFI, semen analysis (sperm density, sperm count, forward motility sperm, sperm motility, etc) will be used as the main indicators of this study, and will be detected at 0th and 8th week.

### 4.2. Secondary outcomes

TCM syndrome score will be used as a secondary indicator in this study, and will be tested at 0 and 8 weeks of treatment.

### 4.3. Safety outcomes

Vital signs (such as respiration, heart rate, body temperature, blood pressure, electrocardiogram, etc), blood routine, urine routine, stool routine, liver function, and renal function will be used as safety indicators, and will be treated at 0, 4, and 8 weeks of treatment. detection. If an adverse event occurs, the clinical investigator will record it in detail on the CRF (including symptoms, time of onset, duration, examination and results). Serious adverse reactions will be reported to the Ethics Committee of the First Affiliated Hospital of Henan University of Chinese Medicine and rescue procedures will be implemented promptly.

### 4.4. Quality control and trial monitoring

All investigators will be trained to ensure study quality before the trial begins. Training covered inclusion and exclusion principles, random sequence generation, allocation concealment, how to intervene with different groups of patients, how to record results and manage data. Clinical investigators responsible for diagnosis and treatment will be registered Chinese medicine practitioners. In order to improve the compliance of the participants, the researchers will conduct health education and fully respect the participants’ right to informed consent. Raw data will be recorded on a CRF and reviewed by 2 data administrators after entering a spreadsheet. To ensure the objectivity of the data, the evaluation and statistics during the trial were blinded. The lead investigator will oversee the entire trial.

### 4.5. Statistical analysis

SPSS 23.0 software will be used by professionals blinded to trial grouping for statistical analysis of data. Efficacy and safety analyses will be based on the intent-to-treat principle of analysis of all randomized participants. Measurement data that conform to normal distribution are represented by mean and standard deviation (^−^*x ± s*), and measurement data that are not normally distributed are represented by median and quartile [M(Q _25_, Q _75_)]. Data are expressed as percentages (%). If the data obey the normal distribution and the variance is homogeneous, the *t* test is used for comparison; if the data does not conform to the normal distribution or the variance is unequal, the nonparametric test should be used for comparison, and the rank-sum test (Mann–Whitney *U* test) is used for the rank data. A 2-tailed test was used, and *P* < .05 indicated a statistically significant difference.

## 5. Discussion

The treatment of male infertility is not easy. Modern medical treatment methods mainly include drugs, surgery and assisted reproduction, but there are limitations such as side effects, high cost and low success rate. TCM has a long history in the treatment of male infertility and is widely used in clinical practice. To a certain extent, it can make up for these limitations and help improve the fertility rate.

Based on the theory that “Sufficient Healthy-Qi inside the body will prevent invasion of pathogenic factors,” from the perspective of male reproduction, Healthy-Qi is a necessary condition to ensure the occurrence and growth of sperm. If the Healthy-Qi is sufficient and the blood is abundant, it can resist pathogenic factors and is conducive to the occurrence and formation of sperm. WWFZYJ is derived from Wuzi Yanzong Pill,^[11]^ which is known as “the first seed recipe in ancient and modern times.” Modern pharmacological studies have shown that astragalus, *Lycium barbarum*, achyranthes, schisandra and psyllium contained in WWFZYJ all contain polysaccharides. Polysaccharides can regulate apoptosis, regulate the function of the immune system,^[12]^ and also protect the reproductive system by scavenging oxygen free radicals and regulating the secretion of hormones by the hypothalamic-pituitary-gonadal axis.^[13]^ Astragalus polysaccharides can promote DNA damage repair through the nucleotide excision repair pathway.^[14]^ *L. barbarum* polysaccharide has a protective effect on spermatogonia oxidative damage, which can improve cell apoptosis, and improve cell viability.^[15]^ Achyranthes polysaccharides can increase the activity of testicular antioxidant enzymes and improve reproductive system function.^[16]^ Schisandra chinensis polysaccharide can improve sperm survival rate and improve testicular tissue damage.^[17]^ Psyllium polysaccharide also has obvious antioxidant capacity,^[18]^ thus protecting the reproductive system.

Plastic pollution has become a global health problem, and the problem of the toxicity of MPs to the reproductive system has also been paid more and more attention. Researches have found that the semen quality and serum testosterone levels of rats exposed to MPs are significantly reduced, the sperm deformity rate is increased, the testicular tissue structure is abnormal, and oxidative stress is active, leading to reproductive dysfunction.^[19,20]^ This shows that MPs pose a great threat to male reproductive health, so it is urgent to find a treatment method for male infertility caused by MPs.

We used MPs content in semen, sperm DFI, semen analysis (sperm concentration, total sperm count, percentage of forward motility, total motility, etc) as the main efficacy indicators, and TCM syndrome score as the secondary efficacy indicators to provide more reference for efficacy evaluation. In addition, vital signs (such as respiration, heart rate, body temperature, blood pressure, electrocardiogram, et al), blood routine, urine routine, stool routine, liver function, and renal function are all safety evaluation indicators. The treatment time is 8 weeks, including follow-up after treatment, so as to better evaluate the prognosis and observe the adverse reactions.

A limitation of this trial is that double-blinding was not possible due to the nature of the intervention. Therefore, we will try to ensure that outcome assessors and data managers are unknown about the allocation scheme. The inclusion and exclusion criteria will be strictly followed to improve the homogeneity of subjects. We hope that this research can provide evidence for observing the effects of WWFZYJ on semen MPs and semen analysis-related parameters in male infertility patients.

## Acknowledgments

We would like to thank all the patients who will participate in the trial and the staff for their support.

## Authors’ contributions

**Conceptualization:** Baojun Ju.

**Investigation:** Xiao Li, Yongtao Zhang.

**Project administration:** Junchao Yao, Luyu Li.

**Writing – original draft:** Miaomiao Ma, Baojun Ju.

**Writing – review & editing:** Miaomiao Ma, Chenming Zhang.
